# Ventricular switch as an alternative to single-ventricle palliation: An attractive strategy but with certain pitfalls

**DOI:** 10.1016/j.xjtc.2024.01.005

**Published:** 2024-01-18

**Authors:** Antonio Gonzalez-Calle, Alejandro Adsuar-Gomez, Felipe Rodriguez-Mora, Amir-Reza Hosseinpour

**Affiliations:** a Congenital Cardiac Surgery Unit, Virgen del Rocio Hospital, University Hospitals of Seville, Seville, Spain; b Congenital Cardiac Surgery Unit, University Hospital of the State of Vaud (CHUV), Lausanne, Switzerland

**Figure:**
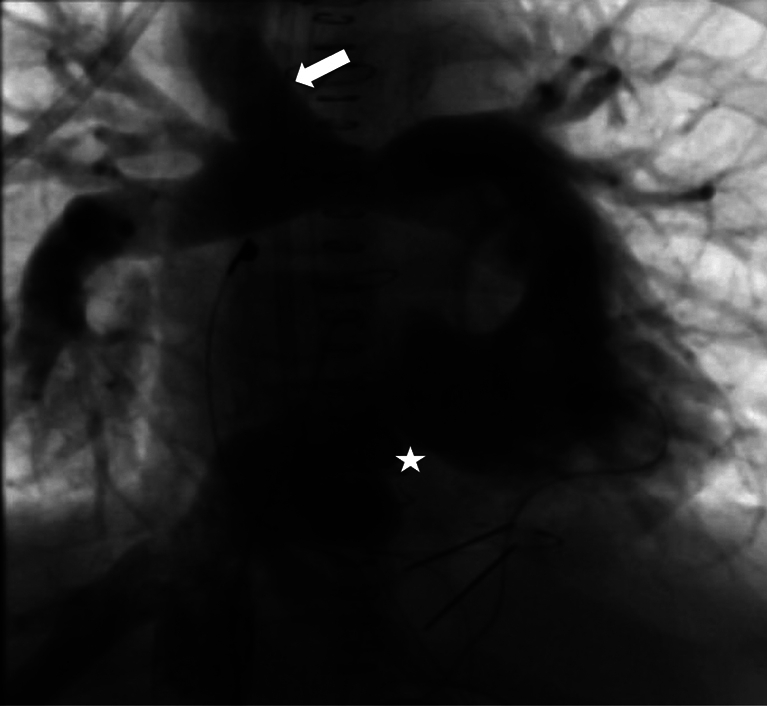
Reverse flow in superior cavopulmonary shunt (*arrow*) and narrow hemi-Mustard path (*star*).

**Central Message**

Ventricular switch is an attractive alternative to single-ventricle palliation, but it does impose certain challenges and pitfalls that should be considered.

Single-ventricle palliation (SVP) imposes significant limitations. This has led to the development of various strategies to push the limits of biventricular repair to avoid SVP. One such strategy, proposed recently, is the concept of ventricular switch (VS).^1^

VS establishes a biventricular circulation in which the morphologic right ventricle (RV) is the systemic ventricle; the exact operation is tailored to each patient according to what is needed to achieve this. Although the RV is not an ideal systemic ventricle, this is considered better than SVP.^1^^,^^2^

VS is proposed in 2 circumstances: (1) to avoid SVP for hearts with 2 good ventricles in which the left ventricle (LV) cannot be connected to the aorta adequately but can to the pulmonary artery (PA), (2) When the LV is not sufficiently developed to support the systemic circulation but may be enough for the pulmonary circulation, although this may require assistance from a superior cavopulmonary anastomosis to achieve a “one-and-a-half ventricle repair.”

VS, being a new concept, is not yet supported with sufficient data. Therefore, all cases ought to be reported for now, especially when complications occur highlighting certain pitfalls of this strategy. We present one such case with permission of the ethics’ committee of the University Hospitals of Seville (reference: 1609-N-23, November 9, 2023) and the patient’s parents.

## Case Report

**Figure 1:**
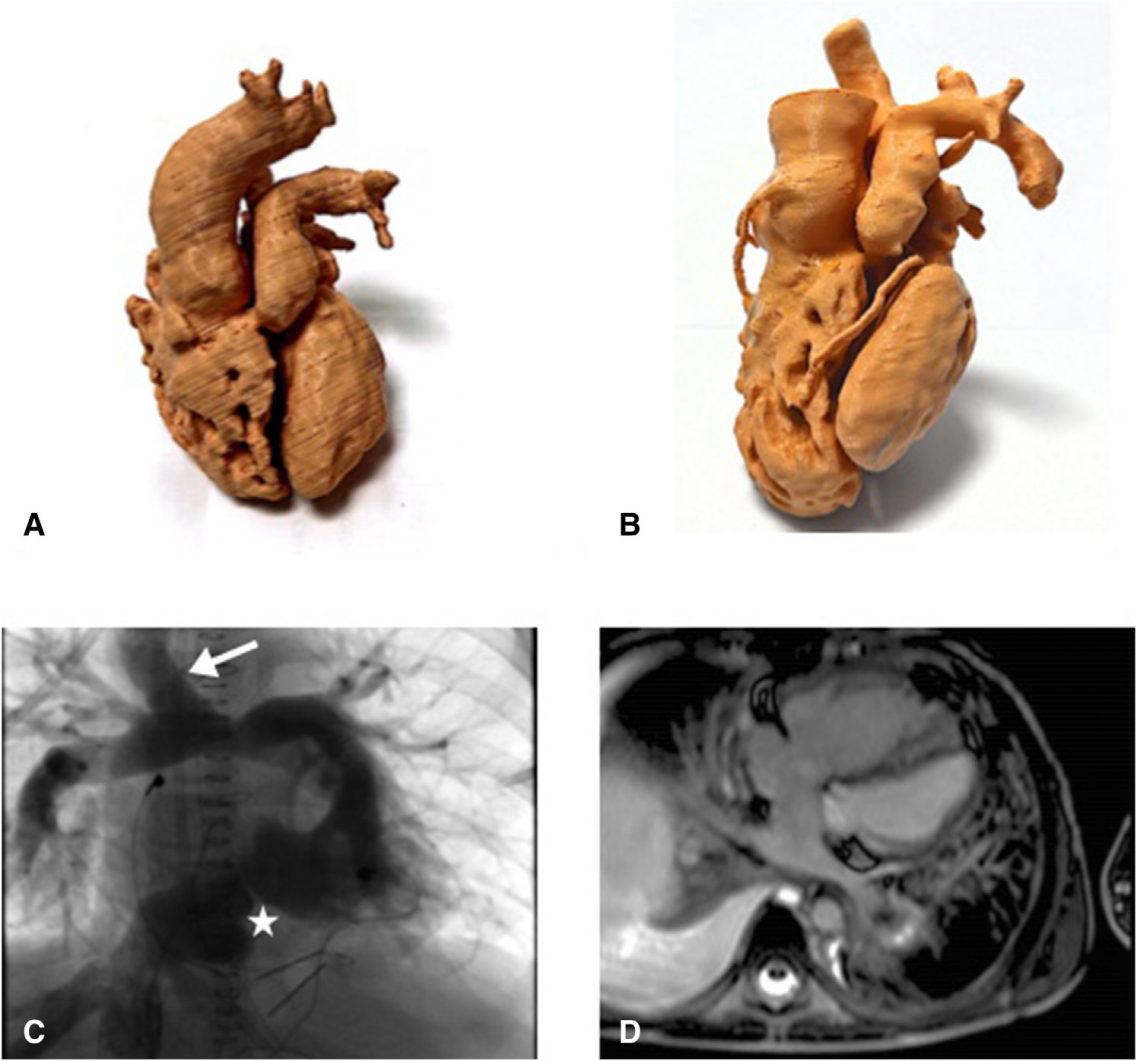
A, Three-dimensional (*3D*) cardiac silicone model, constructed during infancy, showing that both ventricles were well developed. B, 3D cardiac silicone model constructed at the age of 10 years, showing that the left ventricle is noticeably smaller than the right ventricle. C, Angiogram showing flow reversal through the superior cavopulmonary connection (*arrow*) and a narrow hemi-Mustard pathway (*star*). D, Cardiac magnetic resonance imaging, done after the ventricular switch procedure, showing 2 ventricles of normal size.

A 10-year-old boy with double-outlet RV, severe PA stenosis, atrial septal defect, and a noncommitted restrictive muscular ventricular septal defect (VSD) was referred to us with hypoxemia. Biventricular repair had previously been ruled out because the VSD was too small and too far from the ventricular outlets. A superior cavopulmonary anastomosis had been done at 1 year of age. His LV size, having been normal throughout infancy, appeared small (z score <–3) at 10 years of age (Figure 1). His mean pulmonary arterial pressure was 13 mm Hg and pulmonary vascular resistance (PVR) 1.21 U/m^2^ in air. With his parents’ consent, he was offered a VS with a one-and-a-half ventricle strategy: hemi-Mustard, maintaining the superior cavopulmonary connection, VSD closure, pulmonary trunk division and closure, and implantation of a size 14 Contegra conduit connecting the LV to the PA. He was weaned off bypass easily.

He did well initially but then developed pleural effusions, ascites, and hepatomegaly. Catheterization revealed a stenosed hemi-Mustard pathway (gradient: 8 mm Hg), good subpulmonary left ventricle with unobstructed outflow, flow reversal through the cavopulmonary connection, venovenous collaterals, increased mean pulmonary arterial pressure (21 mm Hg) and PVR (3.22 U/m^2^ in air, 1.45 with 85% oxygen), and low end-diastolic pressures in both ventricles (11-12 mm Hg) (Video 1, Figure 1, *C*). Magnetic resonance imaging revealed similar volumes in both ventricles (Figure 1, *D*), and no ventricular dysfunction.

These findings indicated that the LV could receive the entire systemic venous return without increasing its end-diastolic pressure, and that PVR was too high for a cavopulmonary connection. Therefore, the patient was reoperated; the hemi-Mustard and the superior cavopulmonary connection were taken down, and a Senning was done.

The patient did well initially, with cessation of pleural effusions and ascites, and a significant reduction in hepatomegaly. However, 2 weeks later, he developed chest discomfort, followed by cardiac arrest with no response to resuscitation. Autopsy revealed massive myocardial infarction of the hypertrophied systemic RV and subendocardial fibrotic lesions but unobstructed coronary arteries, venous pathways, and Contegra conduit.

## Comments

This case exposes certain pitfalls in the VS concept. First, PVR increased after surgery. This is also seen after heart transplantation in patients undergoing Fontan.^3^ It may possibly be due to the sudden establishment of normal pulsatile flow into a pulmonary vasculature that has received low-pressure nonpulsatile flow for several years and may consequently be chronically partially collapsed with endothelial dysfunction.

Second, assessment of ventricular size to decide between biventricular and one-and-a-half ventricular repair may be difficult. We had misjudged the LV; we could have opted for full biventricular repair from the beginning. In retrospect, the LV appeared small probably only because it was off-loaded.

Third, the systemic RV may be at risk of ischemia due to a combination of hypertrophy, inefficient coronary circulation (having only one coronary artery), and low capillary density relative to muscle mass.^4^^,^^5^ This may be aggravated postoperatively because septating the heart makes the RV lose the assistance of the LV in supporting the systemic circulation (the RV workload increases).

Fourth, there are technical challenges such as surgeons lacking experience in the Senning and hemi-Mustard operations, and the difficulties of implanting a conduit on the LV, which is usually quite leftward and posterior. In conclusion, VS is an appealing concept. Nevertheless, it imposes challenges that should be considered.

## Conflict of Interest Statement

The authors reported no conflicts of interest.

The *Journal* policy requires editors and reviewers to disclose conflicts of interest and to decline handling or reviewing manuscripts for which they may have a conflict of interest. The editors and reviewers of this article have no conflicts of interest.

## Supplementary Data

Video 1 Angiography showing a stenosed hemi-Mustard pathway, good subpulmonary left ventricle with unobstructed outflow, flow reversal through the superior cavopulmonary connection, and venovenous collaterals. Video available at: https://www.jtcvs.org/article/S2666-2507(24)00008-7/fulltext.
