# Supplementary figures and images for: Ventricular switch as an alternative to single-ventricle palliation: An attractive strategy but with certain pitfalls
Source: JTCVS Tech. 2024 Jan 18;24:171–3. doi: 10.1016/j.xjtc.2024.01.005 (PMC11145065; doi:10.1016/j.xjtc.2024.01.005)

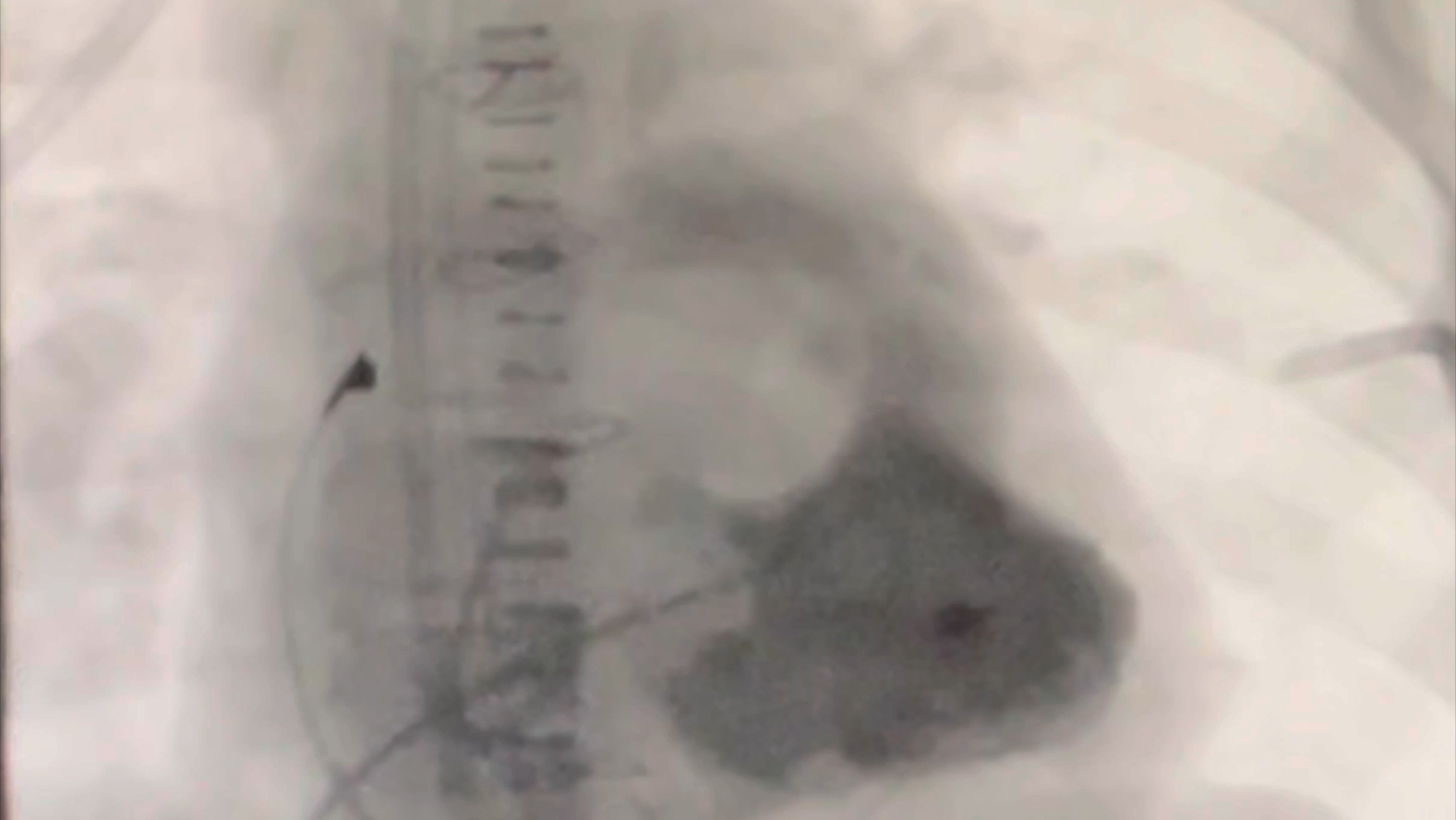

Supplement: Video 1 — Angiography showing a stenosed hemi-Mustard pathway, good subpulmonary left ventricle with unobstructed outflow, flow reversal through the superior cavopulmonary connection, and venovenous collaterals. Video available at: https://www.jtcvs.org/article/S2666-2507(24)00008-7/fulltext. [file fx2.jpg]
